# SYSTEMS LEVEL UNDERSTANDING OF AGING

**DOI:** 10.1093/geroni/igz038.753

**Published:** 2019-11-08

**Authors:** Vyacheslav Labunskyy

**Affiliations:** Boston University School of Medicine, Boston, Massachusetts, United States

## Abstract

Aging is a complex trait is controlled by multiple genetic and environmental factors. Genome-wide screens in invertebrate organisms have led to the identification of hundreds of genes, whose deletion or knockdown can dramatically increase lifespan. Many of these genes represent conserved signaling pathways and biological processes suggesting that they might be involved in the regulation of mammalian aging. Nevertheless, the mechanistic basis by which the majority of these long-lived gene deletions extend lifespan remains poorly understood. Using yeast Saccharomyces cerevisiae as a model system, we employ systems biology network analysis of transcriptomic and protein expression data to build a detailed translational regulatory network of aging in yeast and identify genetic interactions among various aging pathways. Through these studies we expect to gain understanding how the regulatory networks are perturbed with aging, which could provide important insights into human disorders associated with the old age.

